# Size of Openings in Water-Holding Containers: Impact on Oviposition by *Culex (Culex)* Mosquitoes

**DOI:** 10.3390/insects10090257

**Published:** 2019-08-21

**Authors:** Dongyoung Shin, George F. O’Meara, Ayse Civana

**Affiliations:** Florida Medical Entomology Laboratory, Department of Entomology and Nematology, University of Florida-IFAS, 200 9th St. S.E., Vero Beach, FL 32962, USA

**Keywords:** *Culex nigripalpus* 1, *Culex quinquefasciatus* 2, *Culex coronator* 3, oviposition 4, oviposition site selection 5

## Abstract

To assess how a grate covering a catch basin impacts oviposition by *Culex* mosquitoes, a field study was conducted in south Florida using containers with two types of covers, with openings of equal area, but different configurations. One opening mimicked a catch basin grate with 16 small openings, while the other cover had just one large opening. The number and presence of egg rafts in six of each container and cover combination was recorded over 18 nights at two field sites, consisting of a wastewater management facility area and residential subdivision. Three mosquito species, all belonging to the subgenus *Culex* accounted for more than 99% of total egg rafts collected: *Culex* *nigripalpus* (*n* = 1766), *Culex* *quinquefasciatus* (*n* = 754) and *Culex* *coronator* (*n* = 526). Approximately 90% of *Cx. nigripalpus* and Cx. *coronator* egg rafts were deposited in the containers with the large opening cover; whereas more *Cx.* *quinquefasciatus* egg rafts were laid in the containers with small opening covers than those of *Culex* *nigripalpus* and *Culex* *coronator* combined. Similar patterns of egg laying activity were noted at each sampling stations. These results suggest that for locating oviposition sites *Culex* *quinquefasciatus* may rely more on olfactory clues, while other *Culex* species depend more on visual clues.

## 1. Introduction

*Culex nigripalpus* Theobald and *Culex quinquefasciatus* Say are the most common *Culex* (*Culex*) mosquitoes in south Florida where they occur in a wide variety of aquatic habitats, such as, roadside swales, ponds, and numerous types of containers [1,2]. The females of both species will utilize highly nutrient-rich microhabitats as oviposition sites [3,4]. In small natural and man-made containers, for example cemetery vases and tank bromeliads, the immatures of *Culex quinquefaciatus* usually are much more abundant than those of *Culex nigripalpus* [5,6]. When two types of ovitraps were used to assess seasonal activity patterns of four species of *Culex* mosquitoes, *Culex quinquefasciatus* laid approximately equal numbers of egg rafts in both types of traps; whereas *Culex nigripalpus* deposited more than 90% of their egg rafts in the larger of two traps [7]. Identifying the specific factors responsible for trap selection by the two species is difficult because the traps varied not only in size, but also in shape and in the amount of nutrient rich water. 

Containers baited with hay infusion or some other type of attractant are important components of gravid traps [8] and increasing both container and aperture size may result in improved capture rates for some species [9]. Distinguishing the impact of container size versus aperture size on mosquito collections can be problematic since larger containers often have larger apertures [10]. Storm water catch basins are an exception to this general pattern, because mosquitoes often must pass through the rather small openings for the size of each catch basin in the metal grates that cover catch basins. In south Florida, immatures of *Culex quinquefaciatus* are usually much more common than those of *Culex nigripalpus* in catch basins, even though these are relatively large containers [11]. 

The current study was designed to test the hypothesis that container aperture size influences *Culex* oviposition behavior in south Florida. A primary goal of this research was to acquire information that could be used to develop improved gravid traps, particularly for capturing *Culex nigripalpus*. 

## 2. Materials and Methods

**Figure 1:**
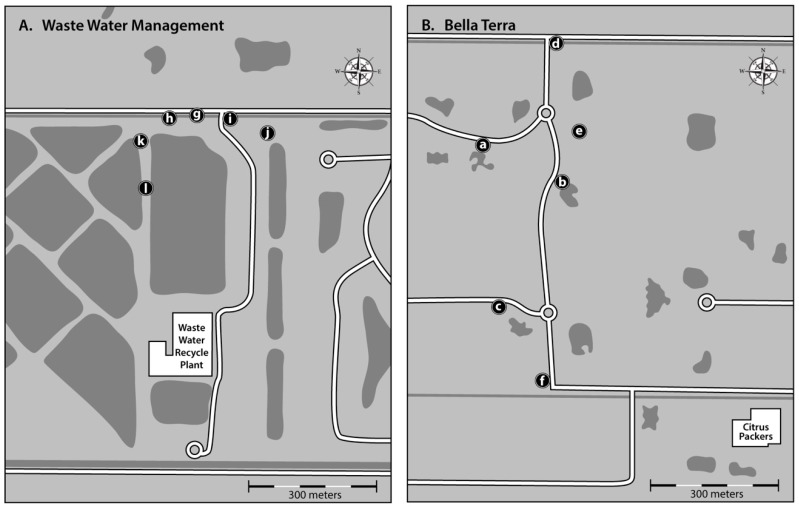
Locations of the oviposition traps: (**A**) Wastewater management facility area; (**B**) Bella Terra: residential subdivision.

Experiments were conducted in Indian River County, FL at two field sites that were recommended by personnel from the Indian River Mosquito Control District. *Culex* (*Culex*) mosquitoes were abundant at both sites during the summer months (Figure 1). Site A was at the county’s west regional wastewater treatment facility wetlands (N 27.617098^0^, W 80.50228^0^) where several ponds with various types of aquatic plants were being used to improve water quality conditions. Site B (N 27.599262^0^, W 80.473899^0^) was in subdivision with many undeveloped lots. Nearby was a citrus processing plant that used a spray field for the disposal of untreated wastewater.

**Figure 2:**
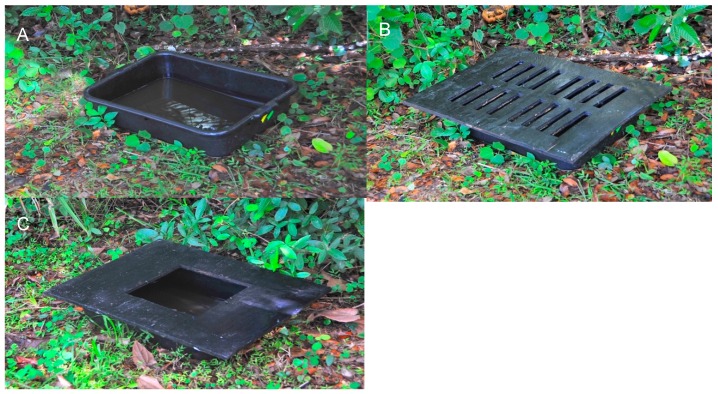
Vats with two different covers for characterizing *Culex* spp. oviposition behavior. (**A**) Hay infusion water in a vat.; (**B**) Ovitrap cover with storm drain type openings; **(C)** Ovitrap cover with a single large opening.

For each site, an ovitrap was placed at six collecting stations that were separated by at least 30 meters. Most of the traps were located in safe, shaded areas to protect them from mowing machines and other types of disturbances. The ovitraps were black plastic vats (47cm L × 35 cm W × 13cm D) covered with a piece of black painted plywood (61 cm L × 48cm W × 1.2 cm D). One type of cover had just one large opening (ca. 600 cm^2^), while the other had 16 small openings (2.5cm L × 15.5cm W), a configuration mimicking what is typically found in grates covering stormwater catch basins (Figure 2). The total amount of opening space in two types of covers was equivalent (ca. 600 cm^2^). Covers were secured to the vats using strips of Velcro. 

To attract gravid *Culex* mosquitoes, ovitraps were baited with 3.8 L of hay infusion, which was prepared with 5 g of hay and 130 mg liver powder/yeast mix (1:6) in 3.8 L of water and held for 3 days before being placed in an ovitrap. New hay infusion was added to the ovitraps just prior to each nightly sampling session. Sampling for egg rafts was conducted from July 15 to August 15, 2014, for total 18 days. For each station, half the ovitrap collections were taken using the cover with the large opening and half of the collections were taken using the cover with small openings. On any given collection date, three ovitraps at each site had a cover with a large opening and three ovitraps had a cover with small openings. Cover types were rotated so that on each successive collection date ovitraps had a different type of cover. 

After each nightly collection, the deposited egg rafts in each vat were transferred individually in vials and stored in a bioclimatic chamber. Once the eggs hatched, the larvae were identified to species using local and regional guides [12,13].

The relationships inter-site variation and ovitrap cover types were analyzed by analysis of variance (ANOVA) to evaluate effects of each independent variable (vat opening types, sites, and time) on the number of egg rafts across three species. 

## 3. Results

We obtained 3059 egg rafts throughout the study belonging to five species of *Culex*, subgenus *Culex*. The collected species were *Culex nigripalpus, Culex quinquefasciatus, Culex coronator, Culex salinarius*, and *Culex interrogator*. Most egg rafts were identified as *Cx. nigripalpus* (*n* = 1768, 57.8%), *Cx. quinquefasciatus* (*n* = 754, 24.6%), and *Cx. coronator* (*n* = 484, 16.4%). Among the egg rafts we collected, were two egg rafts of *Cx. salinarius* and three egg rafts of *Cx. interrogator.* In general, the number of egg rafts varied by opening type, site and date for the most common species (Table 1). Large numbers of egg rafts were collected on some occasions, with maximum numbers of egg rafts from any single container being 34, 164, and 32 for *Cx. coronator, Cx. nigripalpus*, and *Cx. quinquefasciatus*, respectively. 

**Figure 3:**
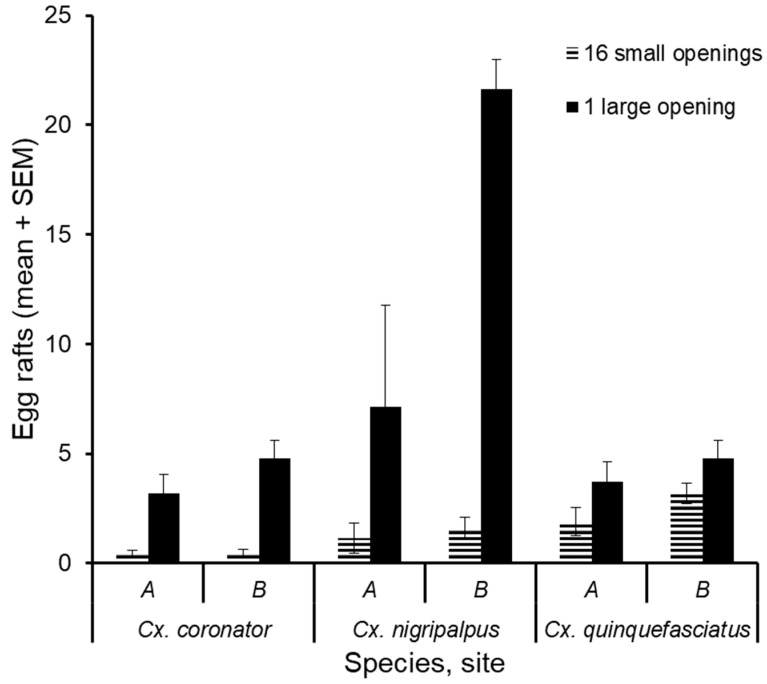
Average number of egg rafts deposited by three *Culex* species in containers with two cover types, at sites A and B. Covers had 16 small openings or one large opening of equivalent total opening area. Bars represent standard error of the mean.

Among these *Culex* species, we found significantly more egg rafts in vats with the large opening (Table 1). The number of egg rafts of *Cx. nigripalpus* was a greater in vats with the single large opening (1614/1768) compared with other *Culex* species (*Cx. quinquefasciatus*: 477/754, *Cx. coronator*: 475/527). We found more egg rafts in vats with the large opening among these *Culex* species. However, the proportion of egg rafts of *Cx. quinquefasciatus* (477/754) in these vats was smaller than the other two *Culex* species (*Cx. nigripalpus* 1614/1768, *Cx. coronator*: 475/527) (Figure 3).

All the *Culex* species sampled during this study laid more egg rafts in the large opening vats (*p* < 0.0001). The oviposition behavior of *Cx. nigripalpus* was not affected by the sites or days, yet *Cx. coronator* was more abundant at site A than site B (*p* = 0.0031) but consistent during the experiment period. The total number of egg rafts at sites A and B was 195 and 289, respectively. Although *Cx. quinquefasciatus* was affected by all the tested factors, cover openings affected this species less than other factors (Table 1).

## 4. Discussion

Two of the five *Culex* species collected during this study are relatively new introductions to Florida. *Cx. coronator* was first detected in the northwest Florida in 2005 [14]. Within a few years it had spread throughout the state [15], occurring in aquatic habitats similar to those utilized by *Cx. nigripalpus.* In 2013, *Cx. interrogator* larvae were collected from a stormwater catch basin in Broward County. More recently, adults and immatures of this species have been collected in three additional counties in peninsular Florida [16]. Mosquito surveillance activities conducted by the Indian River Mosquito Control District during 2014 encountered relatively few *Culex interrogator*, especially when compared to collections of *Cx. nigripalpus* and *Cx. coronator* (Shroyer per. comm.). The scarcity of adult *Cx. interrogator* is the most likely reason why only few egg rafts of this species were collected. *Cx. salinarius* is a common mosquito in north Florida where it is most active during the spring; whereas in the southern part of the state this mosquito is less abundant and usually inactive during the summer months. These factors undoubtedly contributed to the paucity of *Cx. salinarius* egg rafts in our collections.

The land around our two study sites used to be dominated by citrus groves; however, many of them have been taken out of production. The abandoned citrus farms have been turned in pastures or cleared of trees for future residential developments (Figure 1). Due to a high-water table and poorly drained soils, most citrus trees in Indian River County are planted on raised beds usually two rows of trees with a drainage/irrigation ditch in between rows of citrus trees. These ditches often become a major source of mosquito production, particularly following major rainfall events or during an extended dry period when the ditches are flooded for irrigation [17]. Even when groves are abandoned, the ditches often persist, without proper maintenance, in pastures and in undeveloped tracts of land where they continue to provide aquatic habitats for *Cx. nigripalpus*, *Cx. coronator* and other mosquito species. Immatures of *Cx. quinquefaciatus* are not very common in these ditches, probably because the nutrient load in the water is usually not high enough to attract gravid females. Significantly more *Cx. quinquefaciatius* egg rafts were collected at Site B (subdivision: total 441 egg rafts) than at site A (wastewater wetlands: total 300 egg rafts). A spray field near site B was located around Citrus Packers (Figure 1) and receiving untreated wastewater from a citrus processing plant during the winter and spring just prior to the start of the current study (personal communication). This field and some nearby paddocks with small ponds, were likely the major production areas for the *Cx. quinquefasciatus* that deposited egg rafts in the ovitraps a Site B (Figure 1). By contrast, at Site A, the wastewater received a primary and a secondary treatment before being added to the constructed wetlands, hence this aquatic system was essentially unsuitable for *Cx. quinquefasiatus.*

Mosquitoes use both visual and olfactory cues to locate and select suitable oviposition sites [18,19,20,21]. Since all ovitraps contained the same amount of hay infusion and the same square centimeters opening to the outside, it seems reasonable to postulate that olfactory cues emanating from the two types traps were equivalent. Therefore, the preponderance of *Cx. nigripalpus* and *Cx. coronator* egg rafts in the traps with the one large opening relative to the traps with several small openings would seem to result from a visual response by these mosquitoes (Figure 3). Ovitraps with small openings may hinder the mosquito’s capacity to assess the water surface by acting like a blind. As *Cx. quinquefasciatus* prefers nutrient-rich aquatic habitats, it may place a greater reliance on olfactory cues than on visual cues when near a potential oviposition site. Metal grates covering stormwater catch basins probably affect these mosquitoes in a similar manner, because grate covers are unlikely to pose a barrier to *Cx. quinquefasciatus* oviposition.

## 5. Conclusions

In Florida, *Cx. nigripalpus* and *Cx. quinquefasciatus* are the primary vectors of West Nile and St. Louis encephalitis viruses [22,23,24,25]. The recent expansion of *Cx. coronator* populations throughout the state increases the potential for this mosquito to serve as a vector of West Nile virus [26]. Efforts to develop better gravid traps for mosquito and arbovirus surveillance have been focused mainly on various types of infusions and related attractant and the lesser extent other features of the traps [9,27,28,29,30,31]. Generally gravid traps capture far more *Cx. quinquefasciatus* females than *Cx. nigripalpus* females even during the summer months in areas where *Cx. nigripalpus* populations are more abundant than those of *Cx. quinquefasciatus* [29]. Commercially available gravid traps for *Cx.* mosquitoes have rather large collecting devises directly above the water-holding container. By either making these samplers smaller or by redeploying them to the side of the container may allow for enhanced collections of *Cx. nigripalpus* without adversely impacting collections of *Cx. quinquefasciatus*. 

## Figures and Tables

**Table 1:** Effect of cover opening configuration (top), site, and collection day (time) on oviposition in three *Culex* species using ANOVA.

Source	Species	Sum of Squares	Total Number	Prob > F
Opening Type	*Cx. nigripalpus*	9491.3529	28.3308	<0.0001
	*Cx. coronator*	646.04126	33.1189	<0.0001
	*Cx. quinquefasciatus*	138.2409	6.9672	0.0089
Site	*Cx. nigripalpus*	7783.3218	2.1120	0.0209
	*Cx. coronator*	575.73890	2.6832	0.0031
	*Cx. quinquefasciatus*	1724.6655	7.9019	<0.0001
Time	*Cx. nigripalpus*	713.7504	2.1305	0.1459
	*Cx. coronator*	10.85875	0.5567	0.4565
	*Cx. quinquefasciatus*	393.1281	19.8132	<0.0001
